# Zanthoxylum fruit extract from Japanese pepper promotes autophagic cell death in cancer cells

**DOI:** 10.18632/oncotarget.11926

**Published:** 2016-09-10

**Authors:** Reo Nozaki, Toru Kono, Hiroki Bochimoto, Tsuyoshi Watanabe, Kaori Oketani, Yuichi Sakamaki, Naoto Okubo, Koji Nakagawa, Hiroshi Takeda

**Affiliations:** ^1^ Pathophysiology and Therapeutics, Hokkaido University Faculty of Pharmaceutical Sciences, Sapporo, Hokkaido, Japan; ^2^ Center for Clinical and Biomedical Research, Sapporo Higashi-Tokushukai Hospital, Sapporo, Hokkaido, Japan; ^3^ Department of Microscopic Anatomy and Cell Biology, Asahikawa Medical University, Asahikawa, Hokkaido, Japan

**Keywords:** autophagy, autophagic cell death, vacuolization, colon cancer, zanthoxylum fruit

## Abstract

Zanthoxylum fruit, obtained from the Japanese pepper plant (*Zanthoxylum piperitum* De Candolle), and its extract (Zanthoxylum fruit extract, ZFE) have multiple physiological activities (e.g., antiviral activity). However, the potential anticancer activity of ZFE has not been fully examined. In this study, we investigated the ability of ZFE to induce autophagic cell death (ACD). ZFE caused remarkable autophagy-like cytoplasmic vacuolization, inhibited cell proliferation, and ultimately induced cell death in the human cancer cell lines DLD-1, HepG2, and Caco-2, but not in A549, MCF-7, or WiDr cells. ZFE increased the level of LC3-II protein, a marker of autophagy. Knockdown of ATG5 using siRNA inhibited ZFE-induced cytoplasmic vacuolization and cell death. Moreover, in cancer cells that could be induced to undergo cell death by ZFE, the extract increased the phosphorylation of c-Jun N-terminal kinase (JNK), and the JNK inhibitor SP600125 attenuated both vacuolization and cell death. Based on morphology and expression of marker proteins, ZFE-induced cell death was neither apoptosis nor necrosis. Normal intestinal cells were not affected by ZFE. Taken together, our findings show that ZFE induces JNK-dependent ACD, which appears to be the main mechanism underlying its anticancer activity, suggesting a promising starting point for anticancer drug development.

## INTRODUCTION

Cancer is one of the leading causes of death worldwide, and global cancer rates are predicted to increase over the coming years. Chemotherapy is one of the major treatment methods for cancer. Natural products constitute a promising resource for drug development and have always played a key role in pharmaceutical research [1]. Moreover, many single compounds derived from herbs, as well as herbal extracts, have been used clinically to treat various diseases including cancer [2].

*Zanthoxylum piperitum* De Candolle (ZPDC), a deciduous aromatic spiny shrub or small tree native to Japan, is of considerable commercial importance. The dried powder of the pulverized mature fruits of ZPDC, known as ‘Japanese pepper’, is a commonly used spice in Japanese cuisine. Zanthoxylum fruit obtained from ZPDC is also an important component of kampo, a form of Japanese traditional medicine [3, 4]. Previous studies on ZPDC constituents have revealed they can prevent propagation of influenza virus [5], inhibit adipogenesis in an obese mouse model [6], induce vascular relaxation via endothelium-dependent NO-cGMP signaling [7], inhibit cholesterol acyltransferase activity [8], and act as potent tyrosinase inhibitors [9].

In contrast to its effects on other diseases, the anticancer activity of ZPDC has not been widely investigated. The anticancer effects of two different forms of *Zanthoxylum* have been cited in the literature. In one study, an extract from Chinese pepper was shown to inhibit the growth of Neurofibromatosis type 1 (NF1)-deficient malignant peripheral nerve sheath tumor cells by blocking the PAK1/cyclin D1 pathway [10]. In addition, a phytoglycoprotein from Korean ZPDC was reported to inhibit hepatocarcinogenesis [11].

In this study, we tested the anticancer effect of Zanthoxylum fruit extract (ZFE) on four different types of human cancer cell lines (colon, liver, lung, and breast) and then investigated its molecular mechanism of action in the colorectal cancer cell line DLD-1. We found that ZFE causes remarkable cytoplasmic vacuolization in certain types of human cancer cells, leading to the inhibition of cell proliferation and ultimately inducing autophagic cell death (ACD).

## RESULTS

### ZFE induces vacuolization, inhibition of cell growth, and death in cancer cells

**Figure 1:**
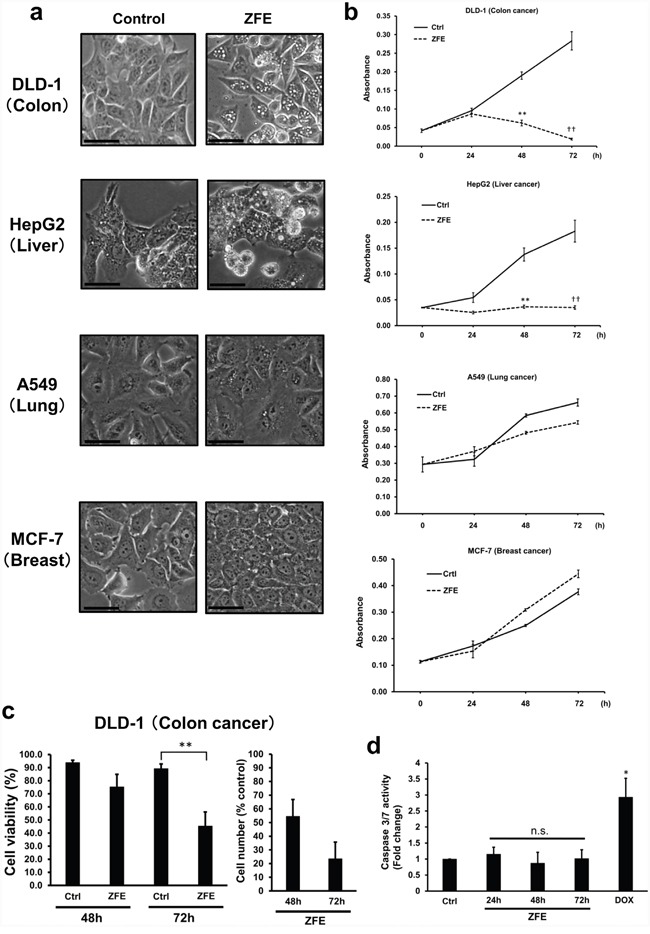
ZFE induces vacuolization and inhibits proliferation in some cancer cells **a.** Effect of ZFE on the morphology of the indicated cells. Cells were incubated with 200 μg/ml of ZFE or 0.2% v/v DMSO (control) for 24 h. Scale bars, 50 μm. **b.** Cells were incubated with 200 μg/ml of ZFE or 0.2% v/v DMSO (control) for the indicated times, and cell viability was measured using a cell proliferation assay kit. Error bars represent S.D. of mean values (n=3). **p < 0.01 vs. control at 48 h; ††*p* < 0.01, vs. control at 72 h (Student's t-test). **c.** DLD-1 cells were treated with 200 μg/ml of ZFE or 0.2% v/v DMSO (control) for the indicated times, and cells were harvested and counted using the Trypan blue exclusion assay. Error bars represent S.D. of mean values (n=3). Cell viability was calculated as the percentage of cells excluding Trypan blue. **p < 0.01 vs. control. **d.** DLD-1 cells were treated with ZFE for the indicated times. DOX treatment for 24 h was used as a positive control for caspase activation. Error bars represent S.D. of mean values (n=3). *p < 0.05 vs. control (Dunnett's test). Ctrl; control, DOX; doxorubicin (7 μM).

First, we investigated the effect of ZFE on the morphology of cancer cells using phase-contrast microscopy. After 24 h treatment with ZFE, numerous vacuoles were observed in the cytoplasm of DLD-1, HepG2, and Caco-2 cells, but not in A549, MCF-7, or WiDr cells (Figure 1a, Supplementary Figure S1a). To determine the effect of ZFE on the proliferation of cancer cells, we performed cell proliferation assays. Proliferation of DLD-1, HepG2, and Caco-2 cells was significantly inhibited after 48 h of ZFE treatment (Figure 1b, Supplementary Figure S1b). By contrast, no inhibition of cell growth was observed in A549, MCF-7, or WiDr cells. Therefore, we investigated the mechanism of the anticancer effect of ZFE in more detail in the human colorectal cancer cell line DLD-1. After 72 h treatment with ZFE, viability and number of DLD-1 cells were reduced by approximately 45% and 25%, respectively, relative to controls (Figure 1c). To characterize ZFE-induced cell death, we assessed markers of apoptosis and caspase-3/-7 activity in the ZFE treated DLD-1 cells. No increase in caspase activity was detected in either ZFE-treated or untreated DLD-1 cells, whereas the cells were able to respond to doxorubicin, an activator of caspase-3/-7 (Figure 1d), suggesting that apoptosis is not involved in ZFE-induced cell death.

### ZFE stimulates autophagy in the colon cancer cell

**Figure 2:**
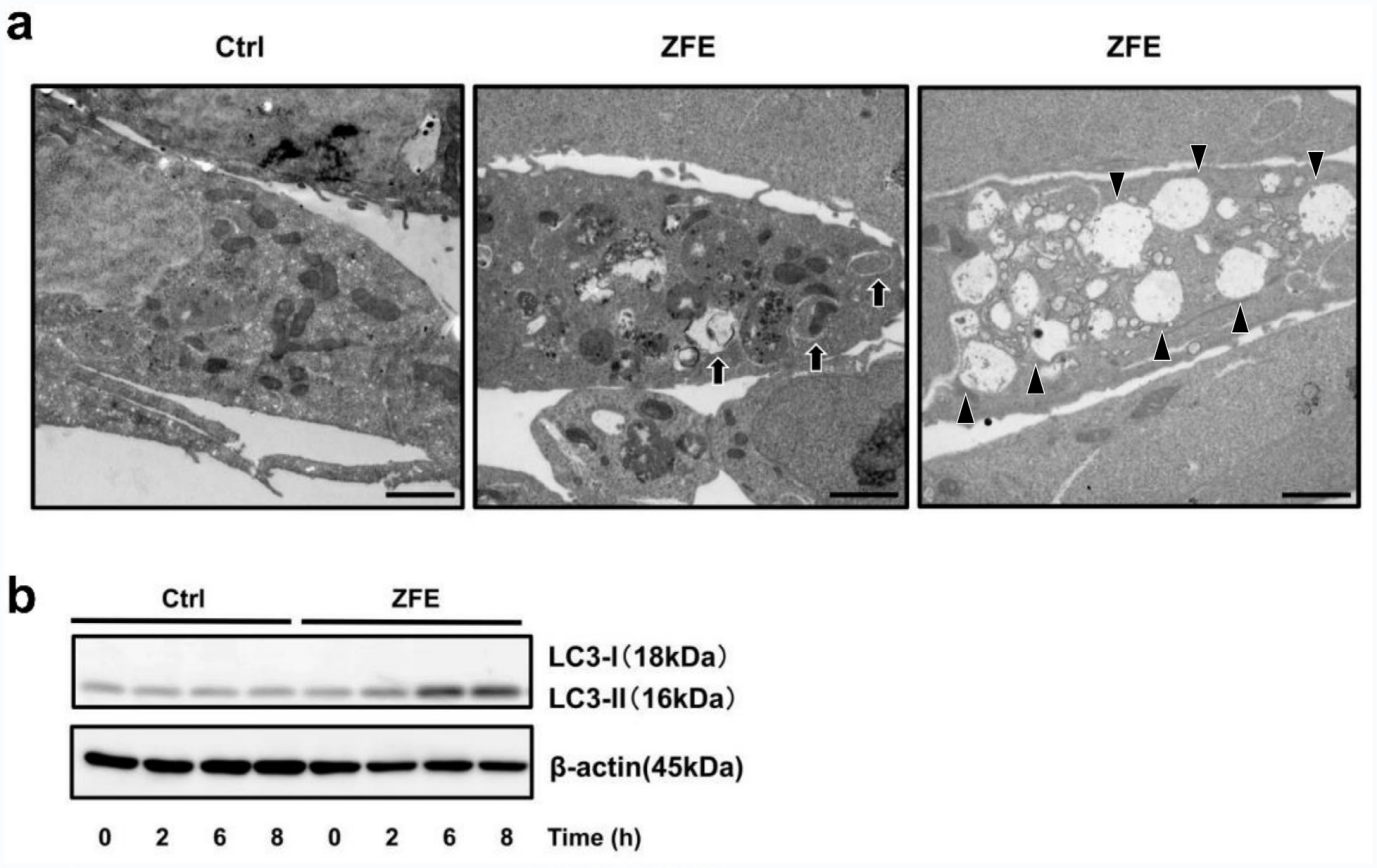
ZFE induces autophagy in DLD-1 cells **a.** Electron micrographs of DLD-1 cells treated with 200 μg/ml ZFE (center and right) or 0.2% v/v DMSO (left) for 24 h. Arrows indicate autophagosomes (center). Arrowheads indicate autolysosomes (right). Scale bars, 2 μm. **b.** Cells were treated with 200 μg/ml of ZFE or 0.2% v/v DMSO (control) for the indicated times. Cell lysates were prepared and subjected to Western blotting with the indicated antibodies. Similar results were obtained in three independent experiments. Ctrl; control.

The occurrence of vacuoles induced by treatment with ZFE was confirmed by electron microscopy (Figure 2a). Electron-microscopic examination of DLD-1 cells after 24 h treatment with ZFE revealed abundant vacuoles and double-membrane structures sequestering cellular organelles, i.e., autophagosomes. These morphological observations strongly suggested that ZFE induces autophagy in DLD-1 cells. To further confirm induction of autophagy by ZFE, we performed Western blotting to analyze the conversion of cytosolic LC3-I into LC3-II in DLD-1 cells. ZFE induced a time-dependent increase in LC3-II levels, confirming elevation of autophagic activity (Figure 2b).

### Knockdown of an essential autophagy protein prevents ZFE-induced ACD

**Figure 3:**
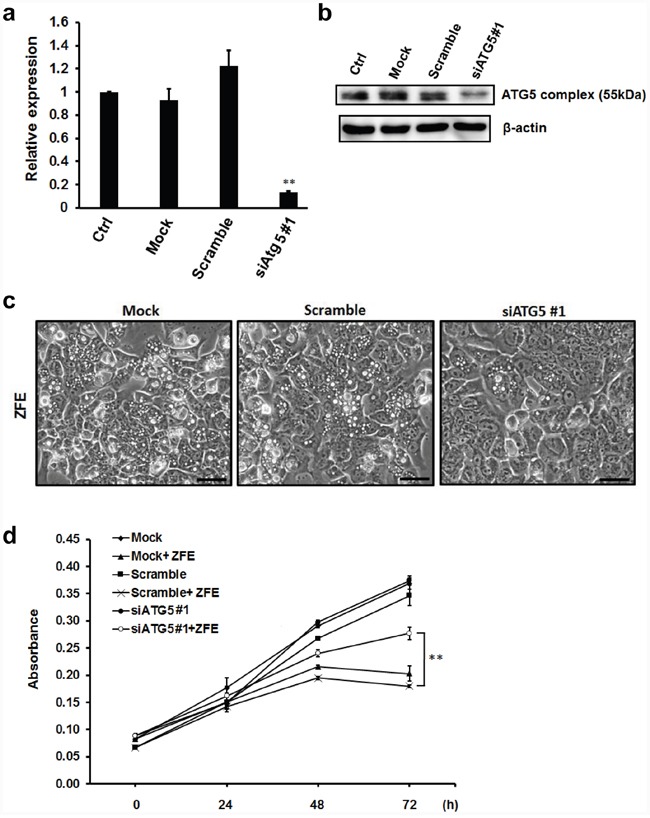
Knockdown of ATG5 protein inhibits the anticancer effect of ZFE in DLD-1 cells **a.** DLD-1 cells were transfected with scrambled siRNA or ATG5 siRNA (10 nM final concentration) or subjected to transfection in the absence of siRNA (Mock). Non-transfected DLD-1 cells were used as control. Twenty-four hours after transfection, RNAs were extracted, and quantitative RT-PCR was performed to measure knockdown efficiency. Fold changes in ATG5 mRNA levels were calculated by the ΔΔCt method, using GAPDH as a reference gene. Error bars represent S.D. of mean values (n=3). **p < 0.01 vs. control (Dunnett's test). **b.** DLD-1 cells were transfected with scrambled siRNA or ATG5 siRNA #1 (10 nM final concentration) or subjected to transfection in the absence of siRNA (Mock). Non-transfected DLD-1 cells were used as controls. Twenty-four hours after transfection, cell lysates were subjected to Western blotting with the indicated antibodies. Similar results were obtained in three independent experiments. **c.** Effect of ZFE on the morphology of ATG5-knockdown DLD-1 cells. Transfected DLD-1 cells were incubated with 200 μg/ml of ZFE for 24 h. Scale bars, 50 μm. **d.** DLD-1 cells were transfected with scrambled siRNA or ATG5 siRNA (10 nM final concentration) or subjected to transfection in the absence of siRNA (Mock). Twenty-four hours after transfection, the cells were harvested and treated with 200 μg/ml of ZFE or 0.2% v/v DMSO (control) for the indicated times. Cell viability was measured using a cell proliferation assay kit. Error bars represent S.D. of mean values (n=3). **p < 0.01 at 72 h (Student's t-test). Ctrl; control.

Autophagy can be inhibited by knocking down the expression of essential autophagy-related genes (ATG). We performed knockdown of ATG5 by transfecting DLD-1 cells with two different small interfering RNAs (siRNAs). Knockdown of ATG5 expression at both the RNA and protein level were confirmed by quantitative RT-qPCR and Western blot analysis, respectively (Figure 3a and 3b, Supplementary Figure S2a and S2b). Autophagic vacuolization induced by ZFE was suppressed by ATG5 knockdown (Figure 3c, Supplementary Figure S2c). Furthermore, the reduction in proliferation following treatment with ZFE was significantly attenuated by knockdown of ATG5 (Figure 3d, Supplementary Figure S2d).

### The role of c-Jun N-terminal kinase activation in ACD

**Figure 4:**
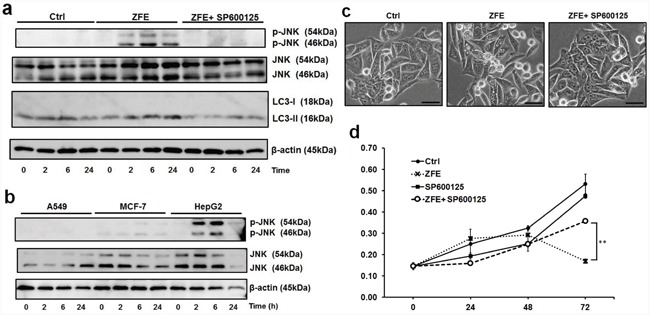
ZFE increases the phosphorylation of JNK, and the JNK inhibitor SP600125 inhibits the anticancer effect of ZFE **a.** Cells were treated with 0.2% v/v DMSO (control) or 200 μg/ml of ZFE in the presence or absence of 5 μM SP600125 for the indicated times. Cell lysates were prepared and subjected to Western blotting with the indicated antibodies. Similar results were obtained in three independent experiments. **b.** A549, MCF-7, and HepG2 cells were treated with 200 μg/ml of ZFE for the indicated times. Cell lysates were prepared and subjected to Western blotting with the indicated antibodies. Similar results were obtained in three independent experiments. **c.** Effect of ZFE on the morphology of DLD-1 cells in the presence of SP600125. DLD-1 cells were treated with the indicated reagents for 6 h. Scale bars, 50 μm. **d.** DLD-1 cells were incubated with 200 μg/ml of ZFE, 5 μM of SP600125, or both for the indicated times; control cells were treated with 0.2% v/v DMSO. Cell viability was measured using a cell proliferation assay kit. Error bars represent S.D. of mean values (n=3). **p < 0.01 at 72 h (Student's t-test). Ctrl; control.

To determine the effect of ZFE on c-Jun N-terminal kinase (JNK), we performed Western blots to measure the phosphorylation of JNK in six types of cancer cells. Phosphorylation of JNK was increased by ZFE in DLD-1, HepG2, and Caco-2 cells, but not in A549, MCF-7, or WiDr cells (Figure 4a and 4b and Supplementary Figure S3a). Next, to explore the role of JNK activation in ZFE-induced ACD, we examined the effects of ZFE on autophagy and proliferation of DLD-1 cells in the presence or absence of the JNK inhibitor SP600125. This compound decreased LC3-II levels and suppressed autophagic vacuolization in ZFE-treated DLD-1 cells (Figure 4a and 4c). Furthermore, the reduction in proliferation following treatment with ZFE was significantly attenuated by JNK inhibitor treatment (Figure 4d).

### Effect of ZFE on the normal rat intestinal cell line IEC-6

**Figure 5:**
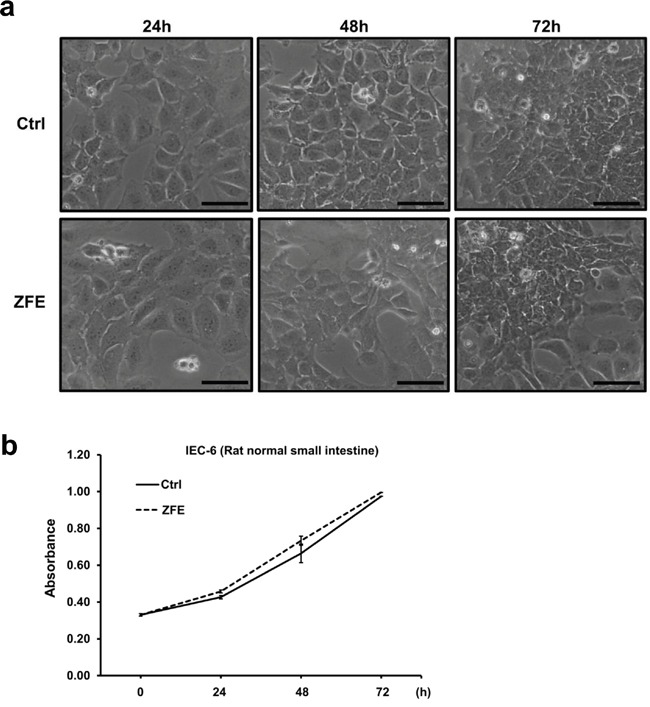
ZFE has no effect on morphology or proliferation of the normal rat intestinal cell line IEC-6 **a.** Effect of ZFE on the morphology of IEC-6 cells. IEC-6 cells were incubated with 200 μg/ml ZFE or 0.2% v/v DMSO (control) for the indicated times. Scale bars, 50 μm. **b.** IEC-6 cells were incubated with 200 μg/ml ZFE or 0.2% v/v DMSO (control) for the indicated times, and cell viability was measured using a cell proliferation assay kit. Error bars represent S.D. of mean values (n=3). Ctrl; control.

To explore the effect of ZFE on normal cells, we treated the rat intestinal cell line IEC-6 with ZFE and examined morphological changes, cell proliferation, and JNK activity. ZFE had no effect on any of these features. (Figure 5a and 5b and Supplementary Figure S3b).

## DISCUSSION

In this study, we found that ZFE induces numerous autophagy-like cytoplasmic vacuoles, inhibits cell proliferation, and induces cell death in certain human cancer cell lines. Furthermore, our results strongly suggest that excess activation of autophagy and JNK-dependent ACD are the main mechanisms underlying the anticancer activity of ZFE.

Autophagy is an intracellular degradation system that delivers cytoplasmic constituents to the lysosome [12, 13]. As such, autophagy plays an important role in the clearance of long-lived proteins and damaged organelles, such as mitochondria, as well as the removal of intracellular pathogens [14].

Cancer cells are likely to be more dependent on autophagy than normal cells [15]. Indeed, basal autophagy activity is elevated in the hypoxic regions of tumors [16]. Cancer cells subjected to stress (e.g., hypoxic environment and/or limited nutrient supply) maximize their energy production, which is required for proliferation, by upregulating autophagy [17].

In some circumstances, excessive activation of autophagy can lead to cell death. ACD, which refers to cell death caused by supraphysiological levels of autophagy, is accompanied by extensive autophagic vacuolization of the cytoplasm and a characteristic vacuolated appearance [18]. It has been proposed that ACD should be regarded as a modality of non-apoptotic and non-necrotic programmed cell death, in which autophagy serves as the mechanism of cell death. Criteria for ACD include the following: (i) cell death occurs without the involvement of apoptosis; (ii) there is an increase in the autophagic flux, not just an increase of autophagic markers, in dying cells; and (iii) suppression of autophagy via pharmacological inhibitors and/or genetic approaches can rescue or prevent cell death [19].

ACD is morphologically defined (especially by transmission electron microscopy) as cell death that occurs in the absence of apoptosis (i.e., chromatin condensation, nuclear fragmentation) or necrosis (i.e., oncosis, swelling of organelles), and is accompanied by the appearance of autophagic vacuoles (i.e., autophagosomes, autolysosomes) in the cytoplasm, resulting in a vacuolated appearance. With this morphological definition in mind, we observed that cancer cells treated with ZFE exhibited an increase in autophagic flux. This observation was confirmed by increased expression of autophagic markers (i.e., LC3-II). Endogenous LC3 is processed posttranslationally into LC3-I, a cytosolic protein. LC3-I (18 kDa) is then converted to LC3-II (16 kDa), which associates with autophagosome membranes [20]. Moreover, the level of LC3-II relative to actin is correlated with the number of autophagosomes per cell [21]. Therefore, ZFE-induced cell death resembled ACD, as determined by morphology and the level of a key autophagic marker.

However, the definition of ACD also requires that cell death can be suppressed by inhibition of the autophagic pathway by treatment with chemicals and/or genetic means (e.g., gene knockout/mutation or RNAi targeting of essential autophagic modulators) [22]. The autophagy-related gene 5 (ATG5)-ATG12 complex, a key regulator of the early autophagic pathway, is required for the formation of autophagosomes. We demonstrated that ZFE-induced cancer cell death and autophagic vacuolization were clearly suppressed by ATG5 knockdown. Thus, ZFE-induced cancer cell death is compatible with the definition of ACD. However, the precise mechanism underlying ACD is still unclear, and the link between autophagy and cell death remains to be elucidated.

Induction of high levels of autophagy may provide a valuable therapeutic strategy for treating cancer. The prospect of establishing novel cancer treatments by modulating autophagy has improved in recent years. Indeed, preclinical studies have been conducted to investigate induction of autophagy and/or ACD for the purpose of cancer therapy [23, 24]. Natural compounds such as resveratrol and curcumin have been implicated in the induction of ACD in various cancer cell lines *in vitro* [24–26]. The findings in these reports are compatible with the observations reported in this study. Thus, ZFE represents a promising starting point for the development of anticancer drugs that act by inducing ACD.

ACD does not simply involve excessive activation of autophagy, but also entails accumulation of ROS and ER stress [27, 28]. We have not yet examined ROS production and ER stress in this context.

The JNK pathway is involved in regulating autophagy of cancer cells in response to various stressors (i.e., starvation, oxidative stress, and pharmacological agents) [29–31]. Moreover, recent studies have indicated that activation of JNK is involved in ACD [32–34]. In this study, we showed that ZFE-induced autophagy and ACD of cancer cells increased JNK phosphorylation. Our results also showed that JNK inhibitor attenuated both ZFE-induced autophagy and ACD in DLD-1 cells. By contrast, ZFE did not exert these effects in normal intestinal cells or other types of cancer cells (i.e., A549, MCF-7, and WiDr). These results indicate that activation of the JNK pathway is required for ZFE-induced ACD.

The JNK pathway is also involved in the induction of apoptosis [35]. However, our results did not support the involvement of apoptosis in ZFE-induced cell death. This observation agreed closely with previous results reported by Shimizu et al., who reported that activation of JNK caused autophagic cell death rather than apoptosis in cells that were already resistant to apoptosis [36].

Taken together, our findings indicate that activation of the JNK pathway is a crucial mechanism of ZFE-induced ACD. Further studies are needed to explore the molecular details of this process.

We also attempted to identify the active component(s) of ZFE. Hydroxy-α-sanshool (HAS) and hydroxy-β-sanshool (HBS) are the most abundant extractable components of ZF, and these compounds have several physiological activities. HAS induces the release and/or production of adrenomedullin, a potent vasodilator peptide hormone, by directly interacting with intestinal epithelial cells [37], and also inhibits two-pore domain potassium (KCNK) channels 9 involved in bowel motility [38]. Based on these findings, we investigated the possible anticancer effect of HAS and HBS on DLD-1 cells. However, no such anticancer activity could be detected for either compound, in contrast to the results obtained using ZFE (Supplementary Figure S4). Therefore, it is unlikely that HAS and HBS are involved in the anticancer effects of ZFE. Further studies are needed to identify the active anticancer component(s) of ZFE.

In summary, we showed that ZFE induces vacuolization and inhibits proliferation of human colorectal and liver cancer cells. Analysis of the anticancer mechanism using DLD-1 cells demonstrated that ZFE activates autophagy, leading to ACD. Taken together, our findings provide novel evidence suggesting that ZFE is a useful tool for analyzing ACD in cancer cells. Moreover, ZFE represents be a promising resource for anticancer drug development.

## MATERIALS AND METHODS

### Reagents and antibodies

Dimethyl sulfoxide (DMSO) was purchased from Wako Pure Chemical Industries Ltd. (Osaka, Japan). Doxorubicin and SP600125 were obtained from Sigma-Aldrich (St. Louis, MO, USA). Hydroxy-α-sanshool and hydroxy-β-sanshool were obtained from Tsumura (Tokyo, Japan). The following antibodies were used for Western blotting analyses: anti-LC3 pAb (PD014), anti-Atg5 mAb (M153-3) (Medical and Biological Laboratories Co. Ltd, Nagoya, Japan), anti-phospho-JNK pAb (Thr183/Tyr185) (#9251S), anti-JNK pAb (#9252S), anti-β-Actin mAb (#4970) (Cell Signaling, Danvers, MA, USA).

### Preparation of the extract from Zanthoxylum fruit

Preparation of Zanthoxylum fruit extract powder, provided by Tsumura (Tokyo, Japan), is described briefly according to Tsumura. The peel of Zanthoxylum fruit was extracted with purified water at 100°C for 1 h. The soluble extract was then separated from the insoluble waste and concentrated by removal of water under reduced pressure. Spray drying was used to generate a dried extract powder. ZFE powder was dissolved in DMSO and sonicated, and the resultant ZFE solution was used to supplement Dulbecco's modified Eagle's medium (DMEM) (Wako Pure Chemical Industries Ltd.) with 3% fetal bovine serum (FBS) (Invitrogen, Carlsbad, CA, USA) and filtered through a 0.22 μm filter unit.

### Cell culture conditions

Human cancer cell lines DLD-1, HepG2, A549, MCF-7, Caco-2, and WiDr and the normal rat small intestinal cell line IEC-6 were cultured in DMEM supplemented with 10% FBS at 37°C in a 5% CO_2_ atmosphere.

### Transmission electron microscopy

DLD-1 cells were grown in 100-mm dishes and treated with 200 μg/ml ZFE or 0.2% v/v DMSO control for 24 h. The cells were fixed with 2% paraformaldehyde–2% glutaraldehyde in 0.1 M phosphate buffer (pH 7.2) for 30 min at 4°C, followed by 1% OsO_4_ for 1 h at 4°C. The cells were gently scraped from the dishes and pelleted in a microcentrifuge. Pelleted cells were then dehydrated with a graded series of ethanol and finally embedded in Epon 812. The ultra-thin sections were cut with an ultramicrotome, contrasted with saturated aqueous solutions of uranyl acetate and lead citrate, and examined using a transmission electron microscope (H-7650; Hitachi High Technologies, Tokyo, Japan).

### Cell proliferation assay

Human cancer cell lines and IEC-6 cells were seeded into 96-well plates at a density of 4 × 10^3^ cells/well and 6 × 10^3^ cells/well, respectively, and incubated overnight to allow cell adherence. The medium was replaced with DMEM (3% FBS) supplemented with 200 μg/ml ZFE and/or 5 μM SP600125 (final concentrations). In negative control samples, the replacement medium consisted of DMEM (3% FBS) supplemented with 0.2% DMSO. Cells were incubated for 24, 48, or 72 h at 37°C under a 5% CO_2_ atmosphere. Proliferation was detected using the CellTiter 96 AQueous One Solution Cell Proliferation Assay kit (Promega, Fitchburg, WI).

### Trypan blue exclusion test

DLD-1 cells were seeded in 12-well plates at a density of 5 × 10^4^ cells/well and incubated for 48 h to allow cell adherence. Subsequently, the cells were treated with 0.2% DMSO or 200 μg/ml ZFE. Cells were incubated for 48 or 72 h at 37°C under a 5% CO_2_ atmosphere. Cells were then harvested, and Trypan blue was added to the cell suspension to a final concentration of 0.2%. The stained cells were counted on a Countess II FL Automated Cell Counter (Thermo Fisher SCIENTIFIC, Waltham, MA, USA). Cell viability was defined as the percentage of live cells (i.e., excluding those staining positively with Trypan blue).

### Caspase-3 and-7 assay

Caspase-3/-7 activities in DLD-1 cells treated with 0.2% DMSO, 200 μg/ml of ZFE or 7 μM doxorubicin were measured using the Caspase-Glo 3/7 assay kit (Promega). The luminescence of each well was measured with a multilabel plate counter (Infinite 200 PRO; Tecan, Männedorf, Switzerland). Luminescence from blank wells (medium without cells) was subtracted. Data were normalized against the luminescence of control cells.

### Western blotting analysis

Cells were treated with 200 μg/ml of ZFE in the presence or absence of 5 μM SP600125 and incubated for 2–24 h at 37°C under a 5% CO_2_ atmosphere. The cells were subsequently washed with ice-cold Tris-buffered saline (TBS) and lysed with 100 μl of cell lysis buffer (25 mM Tris-HCl [pH 6.8], 0.8% w/v SDS, 4% w/v glycerol, 0.008% w/v bromophenol blue, 2% v/v 2-mercaptoethanol). The lysate was boiled at 95°C for 5 min, and then centrifuged at 10,000×g for 5 min. Ten microliters of each sample was loaded onto a 12.5% SDS-polyacrylamide gel; proteins were separated by electrophoresis, and then transferred to Immobilon-P membranes (Millipore, Bedford, MA, USA) by electroblotting. The membranes were immunoblotted with the following antibodies: anti-LC3 pAb, anti-Atg5 mAb, anti-phospho-JNK pAb, anti-JNK pAb, and anti-β-actin mAb. The bound primary antibodies were incubated with horseradish peroxidase–conjugated antibody against rabbit IgG (Jackson ImmunoResearch Laboratories, West Grove, PA, USA) and detected using Immobilon Western HRP Substrate detection reagents (Millipore). Band images were acquired using a LAS 4010 system (GE Healthcare Life Sciences, Amersham, UK).

### RNA interference

Small interfering RNAs (siRNAs) targeting ATG5 (NM_004849) and scrambled siRNA were purchased from QIAGEN (Hilden, Germany). The siRNA target sequences were as follows: ATG5#1 siRNA 5′-AACCTTTGGCCTAAGAAGAAA-3′ and ATG5#2 5′-CTAGGAGATCTCCTCAAAGAA-3′. DLD-1 cells at 60–70% confluence were transfected with a 10 nM final concentration of siRNA using the HiPerFect Transfection Reagent (QIAGEN). Twenty-four hours after transfection, protein and total RNA was extracted.

### RNA extraction and quantitative RT-PCR

Total RNA was extracted using the ISOGEN reagent (Nippon Gene, Toyama, Japan). cDNA was synthesized using PrimeScript RT Master Mix (Takara Bio, Shiga, Japan). Each cDNA (10 ng) was amplified in triplicate using Platinum SYBR Green qPCR SuperMix-UDG (Invitrogen), and then detected on a StepOne Real-Time PCR System (Applied Biosystems, Foster City, CA, USA). Conditions for real-time PCR were as follows: initial incubation at 50°C for 2 min and 95°C for 2 min, followed by 45 cycles of denaturation 95°C for 15 s, annealing at 60°C for 30 s, extension at 72°C for 1 min, and a final round of 95°C for 15 s and 60°C for 15 s and 95°C for 15 s. GAPDH mRNA was used to standardize the total amount of cDNA in real-time PCR. The relative levels of each transcript were determined using the ΔΔCT method, using GAPDH as the control. The primers used for PCR were as follows: ATG5, 5′-TTTGGGCCATCAATCGGAAACT-3′ and 5′-CCACAGGACGAAACAGCTTCT-3′; and GAPDH, 5′-CATGAGAAGTATGACAACAGCCT-3′ and 5′-AGTCCTTCCACGATACCAAAGT-3′.

### Statistical analysis

Statistical significance was analyzed using Student's t-test or Dunnett's test and values of P < 0.05 were considered statistically significant. All data represent results from at least three independent experiments. Error bars represent S.D. of mean values.

## SUPPLEMENTARY FIGURES


