# Supplementary material for: Zanthoxylum fruit extract from Japanese pepper promotes autophagic cell death in cancer cells
Source: Oncotarget. 2016 Sep 10;7(43):70437–46. doi: 10.18632/oncotarget.11926 (PMC5342563; doi:10.18632/oncotarget.11926)
Supplement: Supplementary file 1 [file oncotarget-07-70437-s001.pdf]

Zanthoxylum fruit extract from Japanese pepper promotes autophagic cell death in cancer cells

SUPPLEMENTARY FIGURES

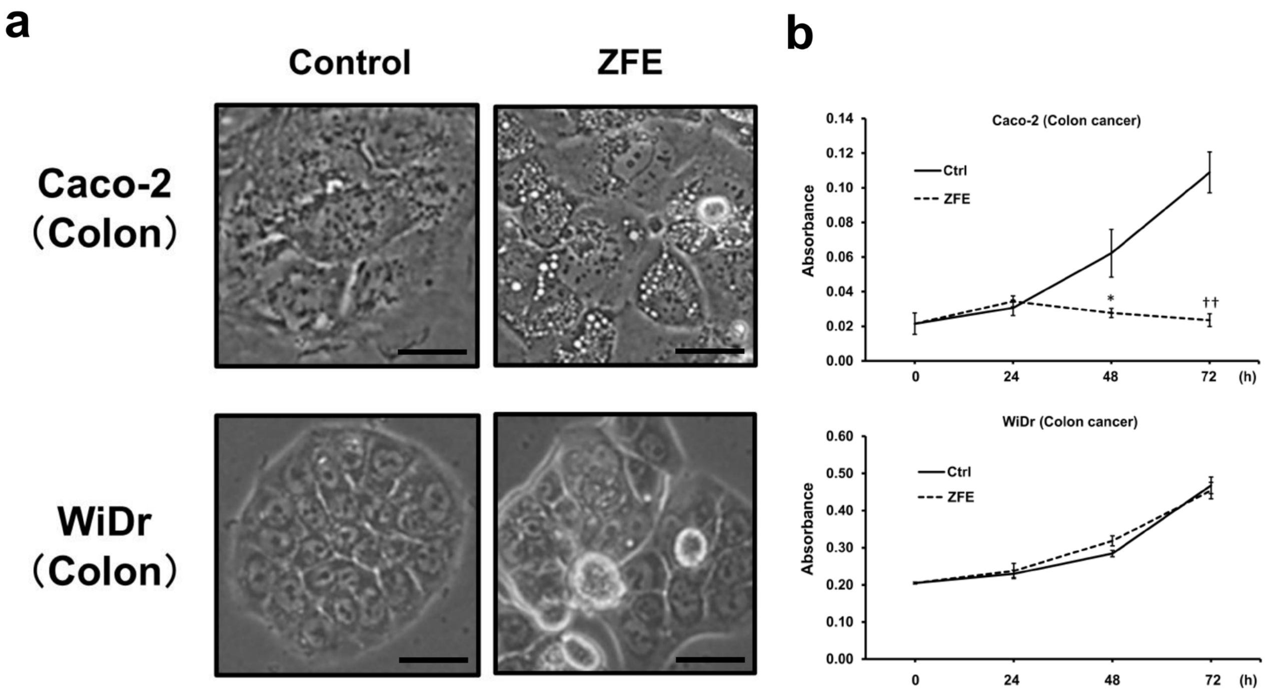

Supplementary Figure S1: Effects of ZFE on morphology and proliferation of human colon cancer cells. a. Effect of ZFE on the morphology of the indicated cells. Cells were incubated with 200 µg/ml of ZFE or 0.2% v/v DMSO (control) for 24 h. Scale bars, 50 µm. **b.** Cells were incubated with 200 µg/ml of ZFE or 0.2% v/v DMSO (control) for the indicated time and cell viability was measured using a cell proliferation assay kit. Data are the mean ± SD of three independent experiments. *$p < 0.05$ vs. control at 48 h. ††$p < 0.01$, vs. control at 72 h (Student's t-test). Ctrl; control.

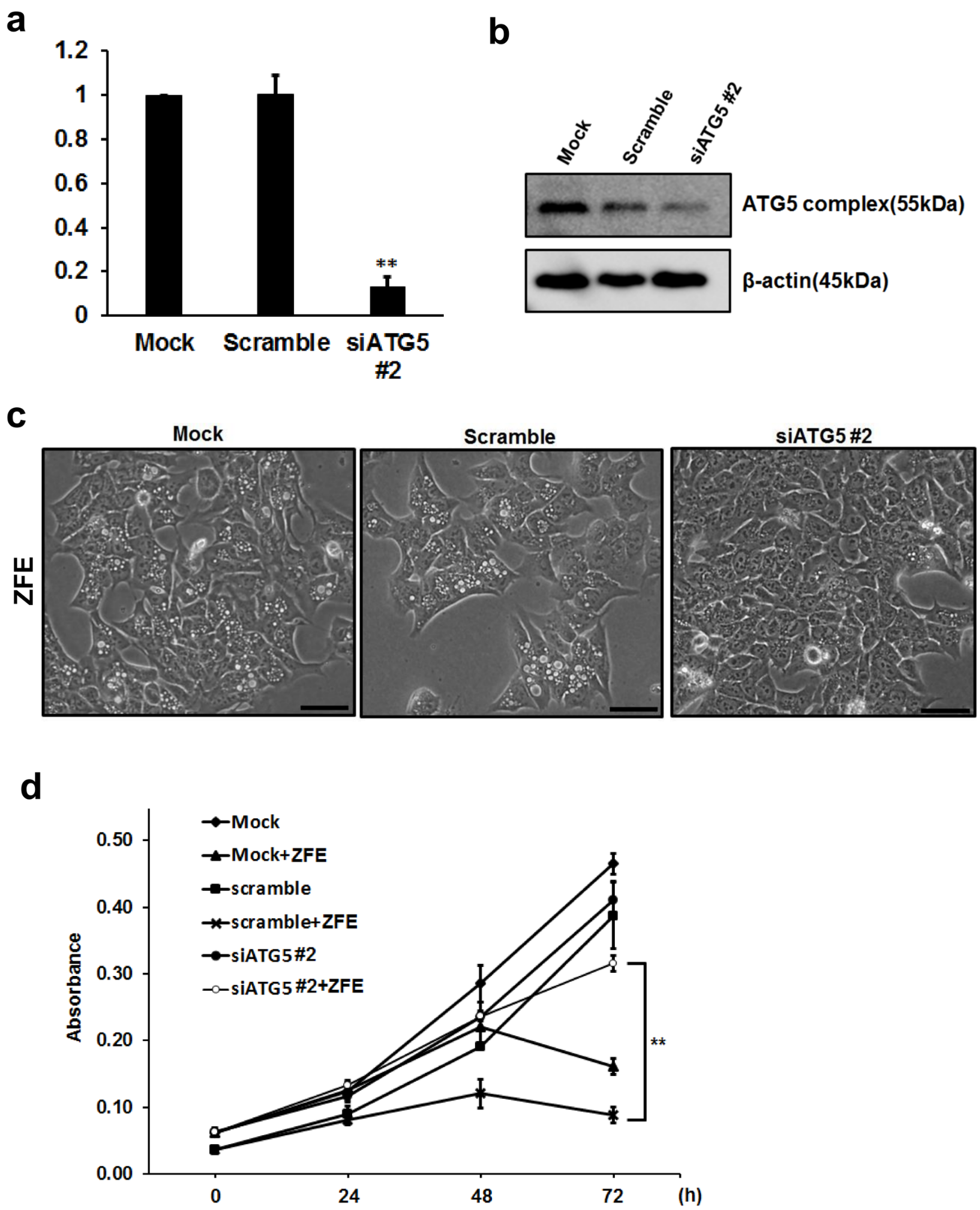

Supplementary Figure S2: Knockdown of ATG5 protein inhibits the anticancer effect of ZFE in DLD-1 cells. a. DLD-1 cells were transfected with scrambled siRNA or ATG5 siRNA #2 (10 nM final concentration) or subjected to transfection in the absence of siRNA (Mock). Twenty-four hours after transfection, RNAs were extracted, and quantitative RT-PCR was performed to the measure knockdown efficiency. Fold-changes in ATG5 mRNA levels were calculated by the ΔΔCt method using GAPDH as a reference gene. Error bars represent S.D. of mean values (n=3). **$p < 0.01$ vs. control (Dunnett's test). **b.** DLD-1 cells were transfected with scrambled siRNA or ATG5 siRNA #2 (10 nM final concentration) or subjected to transfection in the absence of siRNA (Mock). Twenty-four hours after transfection, cell lysates were subjected to Western blotting with the indicated antibodies. Similar results were obtained in three independent experiments. **c.** Effect of ZFE on the morphology of ATG5 knockdown DLD-1 cells. Transfected DLD-1 cells were incubated with 200 µg/ml of ZFE for 24 h. Scale bars, 50 µm. **d.** DLD-1 cells were transfected with scrambled siRNA or ATG5 siRNA #2 (10 nM final concentration) or subjected to transfection in the absence of siRNA (Mock). Twenty-four hours after transfection, the cells were harvested and treated with 200 µg/ml of ZFE or 0.2% v/v DMSO (control) for the indicated time. Cell viability was measured using a cell proliferation assay kit. Error bars represent S.D. of mean values (n=3). **$p < 0.01$ at 72 h (Student's t-test). Ctrl; control.

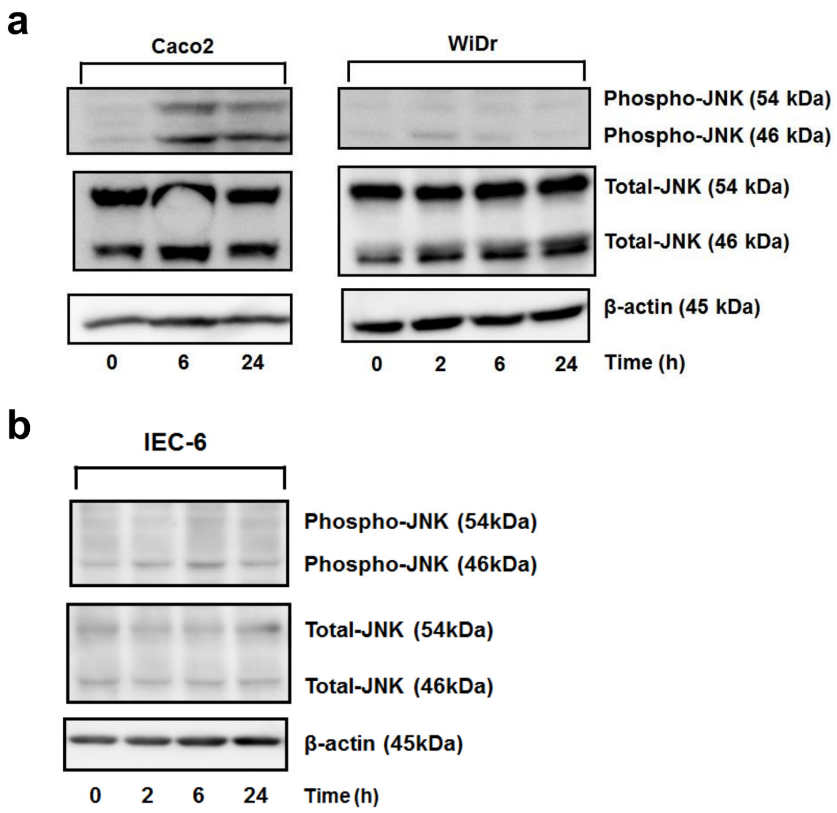

Supplementary Figure S3: Effects of ZFE on phosphorylation of JNK in intestinal cells. a. Cells were treated with 200 µg/ml of ZFE for the indicated time. The cell lysates were prepared and subjected to Western blotting with the indicated antibodies. Similar results were obtained in three independent experiments. **b.** IEC-6 cells were treated with 200 µg/ml of ZFE for the indicated time. The cell lysates were prepared and subjected to Western blotting with the indicated antibodies. Similar results were obtained in three independent experiments.

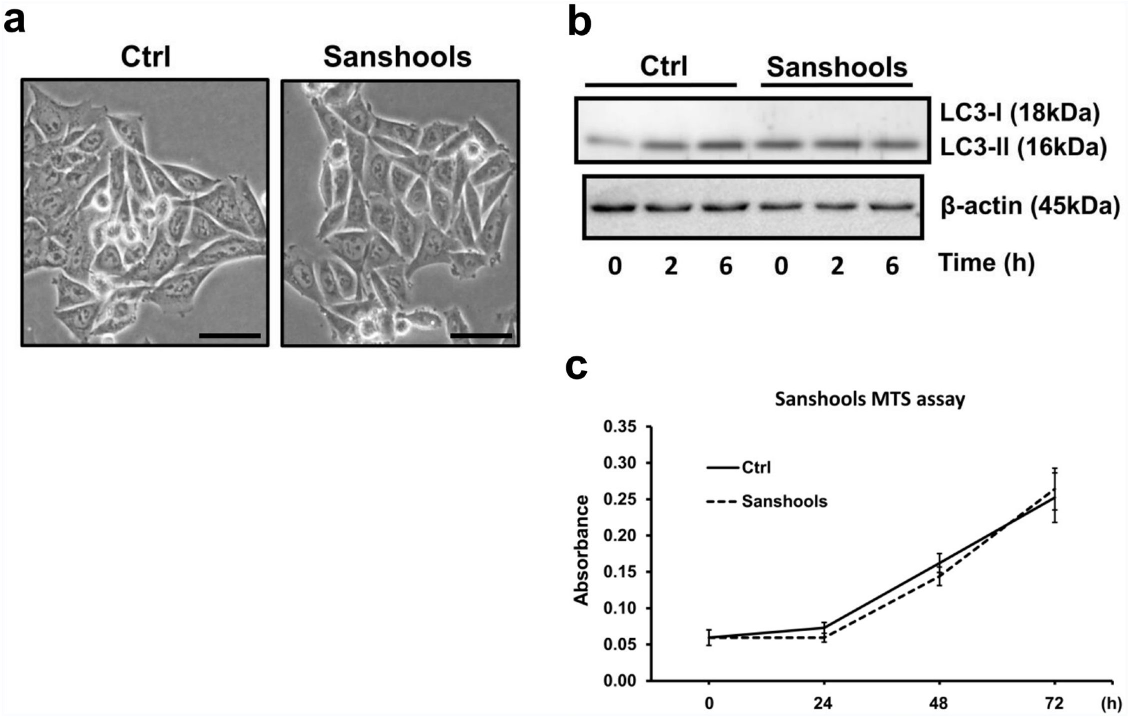

Supplementary Figure S4: Sanshools have no anticancer activity in DLD-1 cells. a. Effect of sanshools (mixture of HAS and HBS at the same concentration) on the morphology of DLD-1 cells. Cells were incubated with 38 µM of sanshools or 0.2% v/v DMSO (control) for 24 h. Scale bars, 50 µm. **b.** DLD-1 cells were treated with 38 µM of sanshools or 0.2% v/v DMSO (control) for the indicated time. The cell lysates were prepared and subjected to Western blotting with the indicated antibodies. Similar results were obtained in three independent experiments.**c.** DLD-1 cells were incubated with 38 µM of sanshools or 0.2% v/v DMSO (control) for the indicated time and cell viability was measured using a cell proliferation assay kit. Data are the mean ± SD of three independent experiments. Ctrl; control.
